# Higher fibre and lower carbohydrate intake are associated with favourable CGM metrics in a cross-sectional cohort of 470 individuals with type 1 diabetes

**DOI:** 10.1007/s00125-024-06213-5

**Published:** 2024-07-05

**Authors:** Douwe F. de Wit, Coco M. Fuhri Snethlage, Elena Rampanelli, Kim Maasen, Noortje Walpot, Daniël H. van Raalte, Max Nieuwdorp, Maarten R. Soeters, Nordin M. J. Hanssen

**Affiliations:** 1 Department of (Experimental) Vascular and Internal Medicine, Amsterdam UMC, Amsterdam, the Netherlands; 2 Department of Endocrinology and Metabolism, Amsterdam UMC, Amsterdam, the Netherlands; 3 Diabeter Centrum Amsterdam, Amsterdam, the Netherlands

**Keywords:** CGM, Macronutrients, Time in range, Type 1 diabetes

## Abstract

**Aims/hypothesis:**

The aim of this work was to investigate the association between macronutrient intakes and continuous glucose monitoring (CGM) metrics in individuals with type 1 diabetes.

**Methods:**

In 470 individuals with type 1 diabetes of the GUTDM1 cohort (65% female, median age 40 [IQR 28–53] years, median diabetes duration 15 [IQR 6–29] years), we used logistic regression to establish associations between macronutrient intakes and the CGM metrics time in range (TIR, time spent between 3.9–10.0 mmol/l blood glucose, optimally set at ≥70%) and time below range (TBR, <3.9 mmol/l blood glucose, optimally set at <4%). ORs were expressed per 1 SD intake of nutrient and were adjusted for other macronutrient intakes, age, sex, socioeconomic status, BMI, duration of type 1 diabetes, pump use, insulin dose and alcohol intake.

**Results:**

The median (IQR) TIR was 67 (51–80)% and TBR was 2 (1–4)%; the mean ± SD energy intake was 6879±2001 kJ, fat intake 75±31 g, carbohydrate intake 162±63 g, fibre intake 20±9 g and protein intake 70±24 g. A higher fibre intake and a lower carbohydrate intake were associated with higher odds of having a TIR≥70% (OR [95% CI] 1.64 [1.22, 2.24] and 0.67 [0.51, 0.87], respectively), whereas solely a higher carbohydrate intake was associated with TBR<4% (OR 1.34 [95% CI 1.02, 1.78]).

**Conclusions/interpretation:**

A higher fibre intake is independently associated with a higher TIR. A higher carbohydrate intake is associated with less time spent in hypoglycaemia, a lower TIR and a higher time above range. These findings warrant confirmatory (interventional) investigations and may impact current nutritional guidelines for type 1 diabetes.

**Graphical Abstract:**

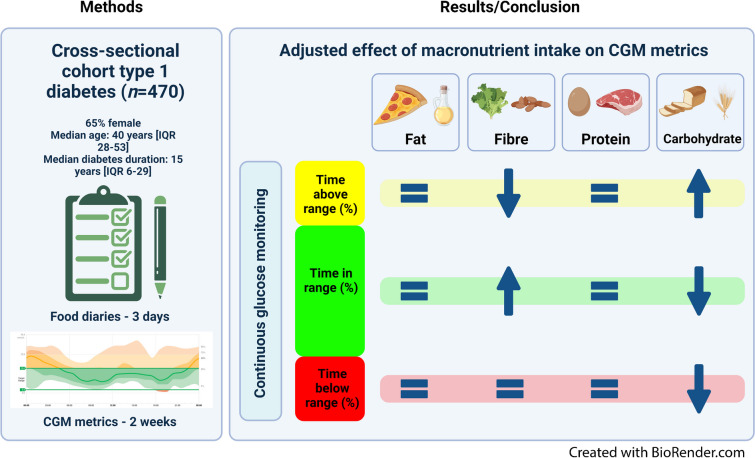

**Supplementary Information:**

The online version of this article (10.1007/s00125-024-06213-5) contains peer-reviewed but unedited supplementary material.


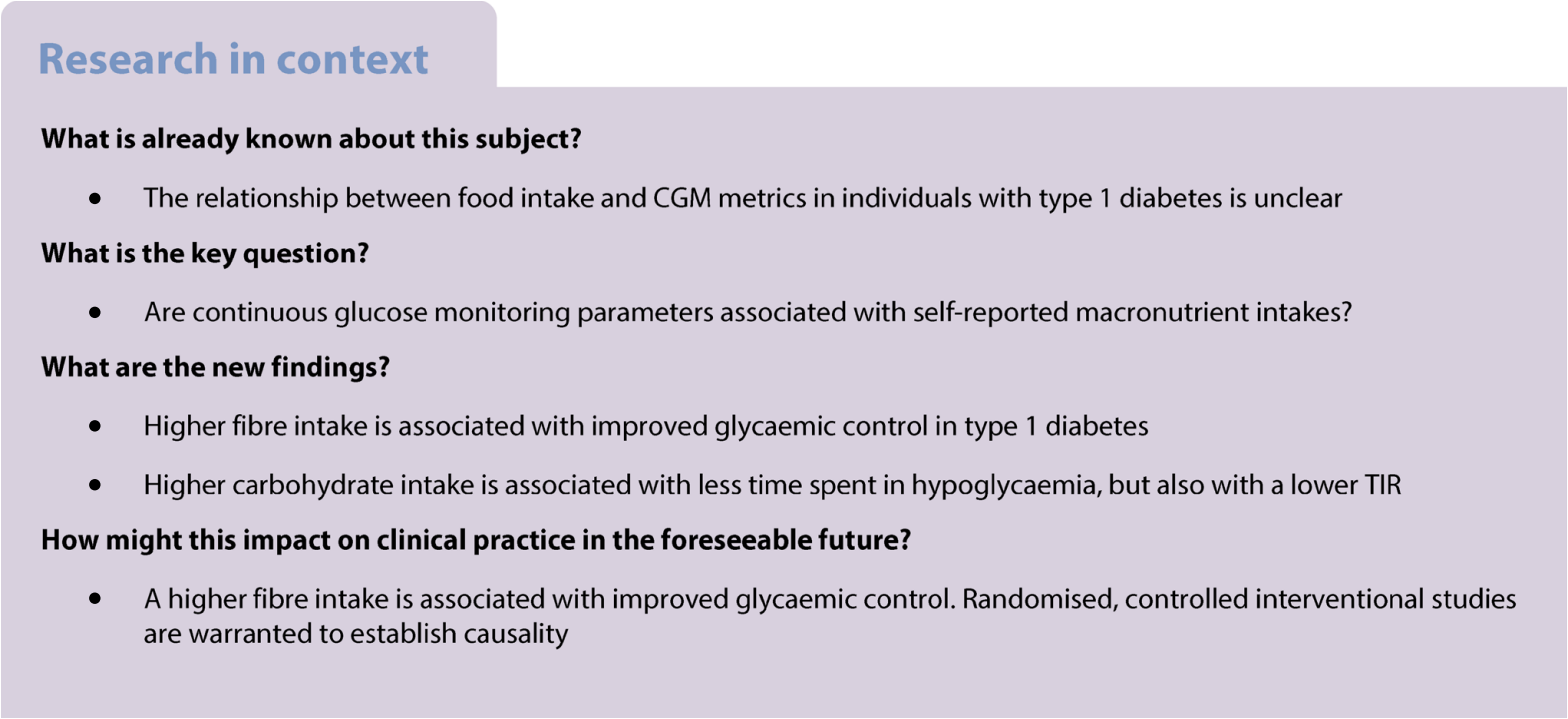


## Introduction

In individuals with type 1 diabetes, continuous glucose monitoring (CGM) has shown consistent benefit as compared with self-monitoring of blood glucose in reducing HbA_1c_ and episodes of hypoglycaemia [1], and has therefore become the standard of glucose monitoring [2]. Time in range (TIR), usually defined as blood glucose between 3.9 and 10 mmol/l, is associated with a lower incidence of microvascular complications [3], and is increasingly used to guide treatment decisions, particularly insulin dose and timing. However, factors beyond exogenous insulin use have a major impact on CGM metrics, including residual beta cell function, as we have recently shown [4]. Therefore, there is a clear need to identify endogenous as well as environmental factors impacting CGM. Here, we investigate nutrition, specifically macronutrient intakes, as a universal yet insufficiently understood factor influencing daily glycaemic control in type 1 diabetes. Although food intake clearly affects glycaemic control, the optimal nutritional pattern for individuals with type 1 diabetes, and specifically its effect on CGM metrics, remains unclear [5]. Current guidelines advise individuals living with type 1 diabetes to consume non-starch-containing vegetables, minimise added sugars and refined grains and choose whole foods over highly processed foods [5] but these recommendations are largely based on the general population. Studies in children and adolescents link increased fibre intake to a lower HbA_1c_ [6] and higher fibre and lower carbohydrate intake to higher TIR [7]. In adults, a small cohort study linked higher carbohydrate intake to favourable CGM metrics [8] but the effect of macronutrients on CGM metrics in adults remain understudied, and hence the optimal nutritional approach in individuals with type 1 diabetes remains uncertain. In this cross-sectional cohort of 470 individuals with type 1 diabetes, we aim to provide robust, independent associations of macronutrients with optimal CGM metrics that may serve as a starting point for future interventional studies and may guide nutritional recommendations.

## Methods

### Study population

The GUTDM1 cohort is a cross-sectional, observational cohort study of 500 individuals with type 1 diabetes that were recruited within the Amsterdam region from 2020 to 2022 as described in detail previously [4]. Potential participants were either contacted by phone by the investigators or asked to participate by their internist or diabetes educator. Participants had to be aged >18 years to participate and the type 1 diabetes diagnosis had to be made by a clinician. Per usual care, individuals with type 1 diabetes received education on medical nutritional therapy and carbohydrate counting by a dedicated type 1 diabetes dietitian. We included individuals with type 1 diabetes regardless of diabetes duration, sex, device use, presence of micro- or macrovascular complications or gastrointestinal conditions such as coeliac disease. Exclusion criteria were active infection at time of inclusion, unwillingness to donate faeces, urine and/or blood, inability to provide informed consent, or absence of large bowel (i.e. colostomy). Participants’ sex was self-reported.

### Data collection

The study consisted of a single hospital visit, where the medical history was reported, a physical examination was performed and fasted blood was withdrawn (for the assessment of HbA_1c_). The day before the hospital visit, participants filled in standardised questionnaires on their insulin use, pump use and real-time CGM (RT-CGM) or intermittently scanned CGM (IS-CGM) device use, as well as exercise (h/week), alcohol consumption (units/week) and smoking status. For 3 days before the visit (two week days and one weekend day), participants recorded data on food intake in an online food diary, using the validated web-based application ‘Eetmeter’ of the Netherlands Nutrition Centre, a Dutch governmental institution on healthy nutrition (eetmeter.voedingscentrum.nl). All food diaries were manually checked for erroneous input and obvious typographical errors were corrected. Two independent clinicians cleaned and verified the data. The daily energy intake of participants was calculated by multiplying the number of grams of a macronutrient by its energy content (37.7 kJ/g for fat, 16.7 kJ/g for protein and carbohydrates, 8.4 kJ/g for fibre). The reported carbohydrate intake included sugar, defined as monosaccharides, but fibre was considered a separate category. CGM data (TIR, time below range [TBR], time above range [TAR], glucose CV [GCV]) during the 2 weeks prior to the hospital visit was collected from the CGM device. Participants used either RT-CGM or at least a second-generation IS-CGM device. In the Netherlands, these devices (FreeStyle Libre, Dexcom, Guardian) are reimbursed for all individuals with type 1 diabetes, so these data were readily available. Participants who did not have a CGM device borrowed one from the investigators for the duration of the study period. Participants were required to have >90% sensor activation during the 2 weeks of data collection. For socioeconomic status, we extracted and applied a publicly accessible composite score of wealth, employment and income per four-digit zip code from Statistics Netherlands (CBS [9]).

## Ethical considerations

The ethical principles of the Declaration of Helsinki were followed and the study was conducted in accordance with the Medical Research Involving Human Subjects Act (WMO). All included participants signed an informed consent form. This study was approved by the medical-ethical committee of the Amsterdam UMC (Dutch central commission of human research number NL73189.018.20).

### Outcomes

TIR, TAR and TBR were calculated by the respective algorithm of the CGM providers and are reported as the percentage of readings showing time spent with blood glucose levels of 3.9–10 mmol/l for TIR, >10 mmol/l for TAR and <3.9 mmol/l for TBR. GCV was calculated by the CGM providers as SD/mean glucose concentration×100 and is expressed as percentage. All of the metrics are reported according to the ADA guidelines [10]. HbA_1c_ was measured in fasted peripheral blood during the study visit, using routine GC–MS in the Amsterdam UMC central laboratory.

### Statistics

Baseline characteristics are presented as mean ± SD or median (IQR), as appropriate for continuous variables, and as percentages for discrete variables. For the nutritional data, extreme outliers were excluded from analyses by erasing the first and 100th percentile of total energy intake (*n*=10), as described previously [11]. Baseline characteristics were stratified to TIR ≥70% or <70%, following the ADA guidelines for optimal TIR [10], and are shown in Table 1. Differences in characteristics by TIR groups were tested using independent samples *t* test for normally distributed variables and Mann–Whitney *U* test for non-normally distributed variables. Spearman correlation was used to correlate individual macronutrient intakes to TIR, TAR, TBR, GCV and HbA_1c_. In a logistic regression model, we expressed the relationship between individual macronutrients and TIR, TAR, TBR and GCV (crude model), and subsequently adjusted for possible confounders as follows: age, sex, diabetes duration, socioeconomic status, BMI, alcohol use, sports (h/week), use of insulin pump (model 1); model 1 + other macronutrients (model 2); and model 2 + total insulin use per day (model 3). Cut-off values of CGM metrics were ≥70% (TIR), ≥4% (TBR), ≥25% (TAR) and ≥36% (GCV), respectively, following current ADA treatment guidelines [10]. Since carbohydrates included sugar, we did not enter sugar separately in the models. To enable comparison between macronutrients, changes in ORs were expressed per SD of macronutrient intakes. Due to collinearity with energy-bearing macronutrients, total energy intake and macronutrients were not tested in the same logistic regression model. Instead, to adjust for total energy intake, we used the ‘nutrition density model’ as described earlier [12], wherein macronutrients are expressed as a percentage of total energy intake, as a sensitivity analysis. We performed the analyses for total daily intake as well as for intake per meal (breakfast, lunch, dinner or snacks). Data with missing values were excluded in the logistic regression model, and missing values per variable are presented in a separate table. An interaction analysis for sex was performed in the logistic regression model. RStudio, version 2022.02.3 was used for all statistical analyses (R Core Team [2021], R Foundation for Statistical Computing, Vienna, Austria).

**Table 1:** Baseline characteristics of study participants, stratified by TIR ≥70% or <70%

Characteristic	TIR<70% (*n*=257)	TIR≥70% (*n*=213)	*p* value^a^
Female sex, *n* (%)	164 (63.8)	141 (66.2)	0.659
Age, years	39.00 (27.00–53.00)	41.00 (29.00–53.00)	0.718
Weight, kg	79.73±15.15	74.66±12.74	<0.001
BMI, kg/m^2^	25.85±4.38	24.68±3.89	0.003
Diabetes duration, years	18.00 (9.00–28.00)	11.00 (3.00–29.00)	0.001
Socioeconomic status, composite score^b^	0.10±0.21	0.13±0.19	0.113
Insulin dose, U/day	42.00 (30.00–57.50)	31.40 (20.00–45.10)	<0.001
Alcohol use, units/day	2.00 (0.00–6.00)	2.00 (0.00–6.00)	0.359
Smoking, *n* (%)	31 (12.1)	14 (6.6)	0.063
Medication other than insulin, *n* (%)	127 (49.4)	92 (43.2)	0.210
Antihypertensive drugs	50 (19.5)	41 (19.2)	0.298
Anticoagulants	14 (5.4)	11 ( 5.2)	0.542
Thyroid medication	33 (12.8)	25 (11.7)	0.515
Other drugs	75 (29.2)	66 (31.0)	0.492
Sensor type, *n* (%)			0.105
Freestyle Libre	195 (75.9)	140 (65.7)	
Dexcom	30 (11.7)	34 (16.0)	
Guardian	23 (8.9)	30 (14.1)	
Pump type, *n* (%)			0.007
No pump	128 (49.8)	103 (48.4)	
Manual	92 (35.8)	58 (27.2)	
Predictive Low-Glucose Suspend	4 (1.6)	1 (0.5)	
Hybrid closed loop	29 (11.3)	38 (17.8)	
DIY closed loop	4 (1.6)	13 (6.1)	
LDL-cholesterol, mmol/l	2.54±0.80	2.58±0.78	0.561
eGFR^c^, ml/min	106.7 (94.9–119.1)	104.4 (91.9–115.8)	0.089
HbA_1c_, mmol/mol	61.28±11.65	49.04±8.62	<0.001
HbA_1c_, %	7.76±1.07	6.64±0.79	<0.001
TIR, %	53.00 (43.00–60.00)	81.50 (76.00–91.00)	<0.001
TBR, %	2.00 (1.00–5.00)	2.00 (1.00–4.00)	0.713
TAR, %	44.00 (36.00–54.00)	14.00 (7.00–21.00)	<0.001
GCV, %	37.70 (32.27–41.90)	30.41 (26.42–34.70)	<0.001
Energy intake, kJ/day	6751.94±1957.40	7031.21±2046.78	0.132
Nutrient intake, g/day			
Carbohydrate intake	167.64 (57.49)	155.92 (67.80)	0.043
Fat intake	70.70 (27.88)	80.06 (33.39)	0.001
Protein intake	67.35 (21.50)	73.03 (26.05)	0.010
Fibre intake	18.73 (8.27)	22.07 (9.58)	<0.001
Sugar intake	62.94 (30.71)	62.44 (32.82)	0.866

Data are presented as mean ± SD, median (IQR) or *n* (%)

Missing values, *n* (%) per variable are as follows: female sex 0 (0); age 0 (0); weight 0 (0); BMI 0 (0); duration 0 (0); socioeconomic status 16 (3.4); insulin dose 2 (0.4); alcohol use 0 (0); smoking 0 (0); medication 0 (0); antihypertensive drugs 2 (0.4); anticoagulants 1 (0.2); thyroid medication 1 (0.2); other drugs 1 (0.2); sensor 18 (3.8); pump type 0 (0); LDL-cholesterol 1 (0.2); eGFR 2 (0.4); HbA_1c_ 2 (0.4); TIR 0 (0); TBR 0 (0); TAR 0 (0); GCV 36 (7.7); energy intake 0 (0); carbohydrates 0 (0); fat 0 (0); protein 0 (0); fibre 0 (0); and sugar 3 (0.6) (see also ESM Table 7)

^a^Mean ± SD values were compared using Student’s *t* test, median (IQR) values were compared using Mann–Whitney *U* test and *n* (%) values were compared using χ^2^ test

^b^The socioeconomic score is a composite of wealth, employment and income per four-digit zip code and is extracted from Statistics Netherlands (CBS); by definition, the average in the Netherlands is 0

^c^By Chronic Kidney Disease Epidemiology Collaboration equation

DIY, do it yourself

## Results

### Study participant characteristics

**Fig. 1:**
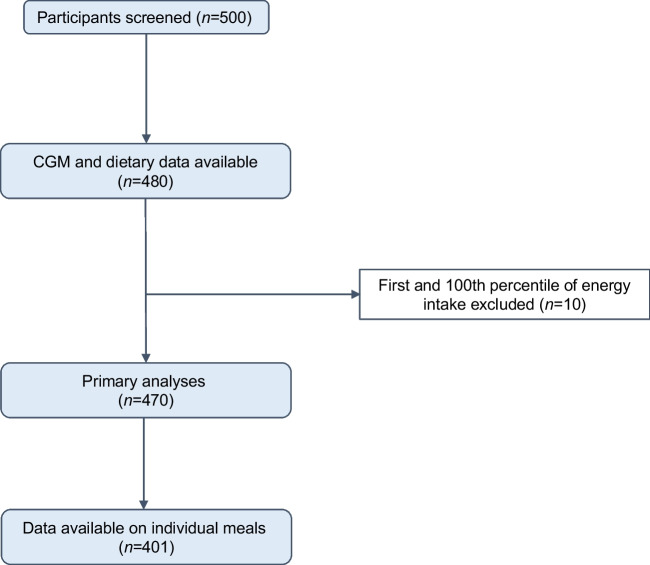
Inclusion flow chart of 470 participants after exclusion of those with missing CGM or nutritional intake data, and those in the first and 100th percentile of energy intake

Out of the 500 GUTDM1 participants, food diaries and CGM data were available for 480 participants (see Fig. 1 flow chart). The upper and lower percentiles of energy intake were defined as outliers and removed (*n*=10), rendering 470 participants available for analysis. Of these, 305 (65%) were female, median age was 40 (IQR 28–53) years and median diabetes duration was 15 (IQR 6–29) years. The median (IQR) TIR was 67 (51–80)% and TBR was 2 (1–4)%; the mean ± SD daily energy intake was 6879±2001 kJ (75±31 g fat, 162±63 g carbohydrate, 20±9 g fibre and 70±24 g protein). In 401 individuals, information on individual meal composition was available. Baseline characteristics for individuals with TIR≥70% and TIR<70% are shown in Table 1 and food intake is summarised in ESM Table 1. Across the cohort, a median of 42 (IQR 34–47)% of total energy intake was accounted for by carbohydrates, 39 (IQR 35–46)% by fat and 17 (IQR 14–19)% by protein, respectively.

### Univariate analyses: food intake association with glycaemic outcomes

Spearman’s correlations between food intake and glycaemic outcomes are shown in ESM Table 2. Fat, fibre and protein intake were significantly correlated with a higher TIR (ρ 0.18, 0.23 and 0.17, respectively) and a lower TAR (ρ −0.17, −0.22 and −0.16), whereas for carbohydrate intake, the opposite effect was observed (TIR ρ −0.15 and TAR ρ 0.16). Accordingly, protein, fibre and fat intake were inversely correlated to HbA_1c_ levels and GCV. None of the macronutrients showed a significant correlation with TBR.

### Multivariate analyses: individual macronutrients linked to favourable CGM metrics

**Fig. 2:**
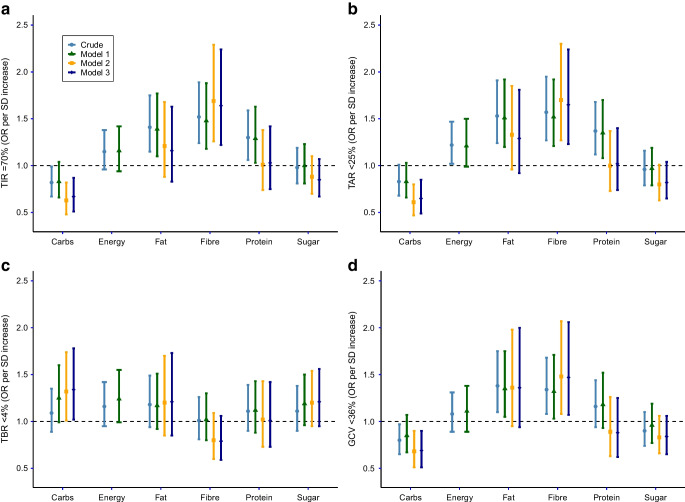
Forest plots of associations of daily energy intake and macronutrient intake with TIR (**a**), TAR (**b**), TBR (**c**) and GCV (**d**). ORs (95% CIs) for favourable CGM outcomes are depicted per SD intake of each macronutrient. One SD represents 63 g carbohydrates, 31 g fat, 9 g fibre, 32 g sugar, or 2001 kJ energy intake. Model 1 adjusted for age, sex, diabetes duration, socioeconomic status, BMI, pump use, exercise and alcohol intake. Model 2, as for model 1, and adjusted for other macronutrients listed. Model 3, as for model 2, and adjusted for daily insulin use. Sugar and carbohydrates are not added in the same model, see ‘Data collection’ in the Methods section. For energy intake, daily insulin use is added to model 1. Carbs, carbohydrates

Next, we used a logistic regression model to investigate the association between total energy intake and individual macronutrient intakes with CGM metrics, when adjusted for each other and for potential confounders (Fig. 2a–d and ESM Tables 3–6). A higher fibre intake was associated with higher odds of favourable CGM metrics, namely TIR ≥70% (OR 1.64 [95% CI 1.22, 2.24]), TAR <25% (OR 1.65 [95% CI 1.23, 2.24]) and GCV <36% (OR 1.47 [95% CI 1.07, 2.06]), but not with TBR <4%. Bedsides fibre intake, protein and fat intake also associated with higher TIR and TAR in the ‘crude’ and adjusted model 1. However, when accounting for other macronutrients and daily insulin usage (model 2 and model 3, respectively) the associations were lost. A higher carbohydrate intake was associated not only with reduced TBR (OR for TBR <4% 1.34 [95% CI 1.02, 1.78]) but also with lower TIR (OR for TIR ≥70% 0.67 [95% CI 1.22, 2.24]), higher TAR (OR for TAR <25% 0.65 [95% CI 0.49, 0.85]) and higher GCV (OR for GCV <25% 0.69 [95% CI 0.51, 0.90]). Total energy intake, and intakes of fat, protein and sugar showed no significant association with any of the CGM metrics after adjustment for possible confounders.

An interaction with sex was not observed in any of the models (*p*_interaction_ >0.05 for all). When expressing carbohydrate, protein or fat intake as a percentage of the total energy intake (the ‘nutrition density model’, [12]), additionally adjusting for sensor type (IS-CGM vs RT-CGM), or adding the upper and lower percentile of energy intake (*n*=10) to the dataset, the associations remained similar (data not shown). Collinearity between the variables in the logistic regression model was excluded (data not shown). When imputing missing values using the median of the variable, the ORs did not change (data not shown). The number of missing values per variable are shown in the footnote of Table 1 and in ESM Table 7.

### Individual macronutrients at meals

**Fig. 3:**
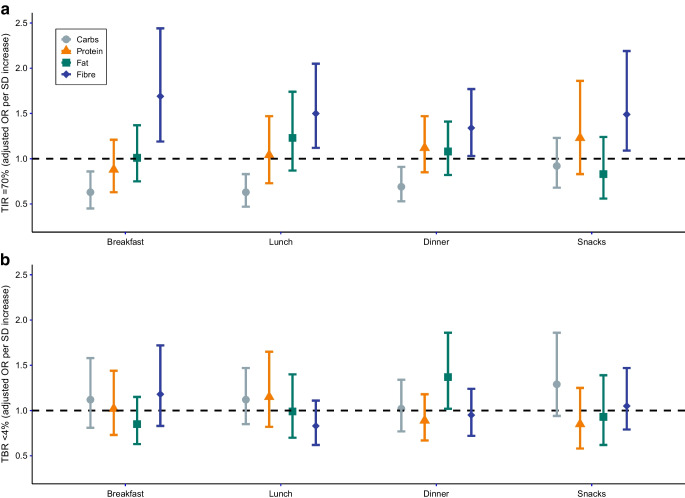
Associations of individual meals with CGM metrics TIR (**a**) and TBR (**b**). Adjusted ORs (95% CIs) for favourable CGM metrics are depicted per SD intake of each macronutrient per meal. For reference SD per macronutrient per meal, please refer to ESM Table 1. All ORs are adjusted for age, sex, diabetes duration, socioeconomic status, BMI, pump use, exercise, alcohol intake, other macronutrients within the same meal and daily insulin use. Carbs, carbohydrates

Next, we assessed macronutrient intakes at different time points (breakfast, lunch, dinner and snacks, as reported by study participants). Overall, we found similar associations for these individual time points as for the overall intake during the day for TIR with largely overlapping CIs for breakfast, lunch, dinner and snacks (Fig. 3). Particularly, fibre intake associated with higher odds of TIR ≥70% at all meals, while carbohydrate intake associated with lower odds of favourable TIR at breakfast, lunch and dinner, but not during snacks. For TBR, macronutrients at individual meals did not show significant associations, except for fat intake at dinner (Fig. 3).

### Between- and within-day variability

To establish the effect of between-day and within-day variability of energy intake on CGM metrics, we calculated a CV (expressed as %) for these parameters. Between-day variability of energy intake correlated weakly (TIR and TAR) or not (TBR and GCV) with CGM metrics using Spearman correlation’s (TIR, ρ −0.11, *p*<0.05; TAR, ρ 0.12, *p*<0.05; TBR and CGV, *p*>0.05). Within-day variability correlated weakly with TIR and TAR (ρ −0.15 and 0.15, respectively, *p*<0.05) and not with TAR and GCV.

## Discussion

In this cross-sectional cohort study, we investigated associations between macronutrient intakes and CGM metrics in individuals with type 1 diabetes. We found that a higher fibre intake is independently associated with a favourable TIR, TAR and GCV. In addition, a higher carbohydrate intake is associated with elevated blood glucose concentrations, as reflected by a lower TBR, a lower TIR and a higher TAR. For the other macronutrients and total energy intake, we found no significant associations with CGM metrics in multivariate analyses. We found no difference in individual meals or between-day or within-day variability with regard to their contribution to glycaemic control, besides fat intake at dinner and TBR (which we consider likely a chance finding).

The vast and rapid implementation of CGM devices has led to a great expansion of data on individual glycaemic excursions in individuals with type 1 diabetes. Subsequently, factors other than exogenous insulin that influence glycaemic control and hence macro- and microvascular complications are increasingly identified. We recently showed that residual beta cell function (as determined by stimulated urinary C-peptide) is a determinant of TIR [4], and others have identified factors including scan frequency [13] and use of non-insulin glucose-lowering medications including but not limited to sodium–glucose cotransporter 2 (SGLT2) inhibitors [14]. The association between higher fibre intake and a lower HbA_1c_ has long been recognised [15] and in 2000 an interventional study also showed that a 24 week high-fibre diet (36 g vs 15 g/day) reduced mean glucose, HbA_1c_ and the frequency of hypoglycaemic events in type 1 diabetes [16]. In another trial, 24 weeks of high-fibre intake (50 g/day) was shown to decrease fasting glucose levels when compared with low-fibre intake (15 g/day) in 61 individuals with type 1 diabetes, although no significant effect on HbA_1c_ was observed [16]. Additionally, there is a considerable body of mechanistic evidence of fibre contributing to improved glycaemic control [17, 18]. However, little evidence links nutrition to CGM outcomes and, to our knowledge, the association of fibre intake with favourable CGM metrics is a novel finding. To date, no clinical trials on the effect of fibre intake on CGM metrics have been performed in type 1 diabetes. Thus, the observed associations of fibre with CGM metrics indicate that addition of fibre may be a valuable tool to optimise TIR in type 1 diabetes and pave the way for an intervention study to confirm this hypothesis. In line with our data, a previous cross-sectional study in adolescents and children with type 1 diabetes disclosed that the participants with TIR ≥70% followed a diet containing more fibre and protein than participants with TIR <70% [7]. Of note, only 31% of our cohort met the fibre intake levels recommended by the ADA (3.4 g/MJ, [19]), supporting the hypothesis that increasing fibre intake will increase TIR. Dietary fibres are a heterogeneous group and their effect on glycaemic control depends on their molecular weight [20], viscosity [20] and interplay with co-consumed foods [21]. Since it is challenging to extract the type of fibres consumed from food diaries, interventional trials are needed to establish specific effects on GCM metrics. In the meantime, messages to increase fibre consumption from whole grains, legumes, vegetables, fruits and nuts can be broadly supported by food and nutrition practitioners regardless of impact on glycaemia [22].

For carbohydrates, a higher intake was associated with a lower TBR, lower TIR and higher TAR, suggesting a general increase in glucose levels with increasing carbohydrate intake. It thus appears that carbohydrate restriction may aid in glycaemic control but at the cost of hypoglycaemia. Indeed, a previous report showed improved TIR but an increased incidence of hypoglycaemic events in a small cohort of individuals consuming very low amounts of carbohydrate (28 g/day) [23]. On the other hand, a low-carbohydrate intake (47 g/day) led to less time spent in hypoglycaemia than an intake of 225 g/day in a 1 week trial in ten individuals [24], a finding replicated with a low-carbohydrate intake of 98 g/day in another 12 week trial [25]. Mismatching insulin administration to carbohydrate intake may explain these heterogeneous results, since this may lead to hypo- as well as hyperglycaemia regardless of changes in carbohydrate intake. Our findings, adjusted for total daily insulin use, suggest an optimal amount of carbohydrate intake that is low enough to increase TIR but high enough to prevent hypoglycaemia. Larger intervention trials are needed to quantify this optimal amount, which may be dependent on many factors (e.g. basal metabolism, physical activity and meal composition). In real life, macronutrients are not consumed in their pure form, and increasing the intake of fibre-rich foods, such as beans, fruits or whole grains, will often result in increased carbohydrate intake. In this regard, the glycaemic index (GI) of various carbohydrates may be an important determinant of glycaemic control, since low GI meals diminish postprandial glucose excursions, at least in paediatric studies [26, 27]. However, there is limited evidence regarding longer low-GI nutritional patterns in individuals with type 1 diabetes and in our dataset, we did not account for different types of carbohydrates. The obscure role of carbohydrate intake in glycaemic control is reflected in the ADA guidelines, wherein the optimal amount of carbohydrate intake for individuals with type 1 diabetes is currently not defined [5].

In our study, we observed no effect of fat intake on CGM metrics and it thus appears reasonable to replace carbohydrates with unsaturated fat as an energy source, in line with the ADA guidelines, which support the use of unsaturated fats [19]. However, the effects of such a Mediterranean diet (high in unsaturated fats and fibre, low in carbohydrates) have not been tested in trials in individuals with type 1 diabetes and thus further research is warranted.

The timing of food intake has been studied extensively in the past decade and, in general, later rather than earlier intake of the main meal of the day is associated with worse insulin sensitivity and decreased postprandial thermogenesis [28]. In type 1 diabetes, the extent to which individual meal quantity, timing and composition affects glycaemic control remains largely obscure. A Finnish study showed that around 40% of energy intake takes place between 18:00 and 23:59 hours, and associated a higher number of meals with a better glycaemic control [29]. However, this study did not use CGM metrics and thus did not allow observation of the effect of specific meals on CGM data. The associations we observed in different meals largely reflected those we found for total daily intake, suggesting that timing of food intake is inferior to macronutrient composition. The fact that we could not see the ‘morning’ effect (a favourable effect of earlier food intake) in our population could be due to the fact that endogenous insulin, which has a circadian rhythm [30], may play a less important role in individuals with type 1 diabetes. However, we were not able to detect ‘subtle’ effects (e.g. lunch at 12.00 or at 15.00 hours) in our dataset. Therefore, we cannot fully rule out the effect of a circadian rhythm on CGM metrics.

### Strengths and limitations

The major strengths of this study are the size of the study population, allowing for robust associations of macronutrients with CGM metrics. Furthermore, the study design enabled a comprehensive adjustment for possible confounders, such as pump type, socioeconomic status, insulin use and the number of hours spent on physical exercise. Third, this study addresses an important knowledge gap since the effects of nutritional habits on CGM metrics are clinically relevant but remain understudied. Since we included adults with type 1 diabetes regardless of diabetes duration, sex or complications, we think that these findings are generalisable to the adult population with type 1 diabetes in the Netherlands. In addition, we observed no significant interaction between sexes, supporting generalisability.

This study also has several limitations. Due to the cross-sectional design, we provide associations rather than causal relationships, and because the CGM data and food diaries were not per se collected on the same day, we could not measure the direct effect of macronutrients on glucose concentrations. Although we used a validated nutrition diary, underreporting is a common problem [31] and we therefore argue that the associations between fibre and carbohydrates and CGM metrics are if anything an underestimation. Reporting bias may occur since participants might either eat more desired food on the reporting days or report desired rather than actual food intake. Furthermore, although we are cautious about fully excluding associations between fat and protein intake and CGM metrics, our investigation argues for the stronger effect of fibre and carbohydrate over fat and protein intake on CGM metrics as underreporting is likely to occur across all nutrient groups. Information on socioeconomic status, which is associated with glycaemic control in type 1 diabetes, is not taken into account [32]. In addition, we miss reporting on snack frequency and timing over the day; therefore, we cannot interrogate how small meal intervals influence the impact of individual macronutrients on glycaemic outcomes. Variation in timing of meal-time insulin is not reported; however, we argue that if anything this phenomenon underestimates the association of nutrition on CGM outcomes, and it therefore does not weaken our findings. Likewise, the median TIR in our cohort was 67%, which is considerably higher than in other cohorts [33, 34], although we argue that this only underestimates the association of higher TIR with fibre intake. It is likely that individuals with a higher fibre intake will eat healthier in general. Although we took this into consideration by adjusting for intakes of other macronutrients, future observational studies may adjust for general diet quality, by applying quality indices such as the Healthy Eating Index, which has been associated with HbA_1c_ in earlier work [35].

### Conclusion

In conclusion, dietary fibre intake is consistently associated with favourable CGM metrics in individuals with type 1 diabetes. The role of carbohydrates is more complex, since carbohydrate restriction may lead not only to more TIR but possibly also to more TBR. Our data indicate that a high-fibre, low-carbohydrate nutritional pattern favours glycaemic control in individuals with type 1 diabetes. These findings warrant confirmation in interventional studies and may contribute to more tailored nutritional lifestyle advice to improve glycaemic control in type 1 diabetes.

## Supplementary Information

Below is the link to the electronic supplementary material.

Supplementary file1 (PDF 870 KB)

## Data Availability

The data that support the findings of this study are not openly available due to reasons of sensitivity and are available from the corresponding author upon reasonable request.

## Abbreviations

CGM: Continuous glucose monitoring
GCV: Glucose CV
GI: Glycaemic index
IS-CGM: Intermittently scanned CGM
RT-CGM: Real-time CGM
TAR: Time above range
TBR: Time below range
TIR: Time in range

## Authors’ relationships and activities

MN is a scientific advisory board member of Caelus Pharmaceuticals and Advanced Microbiome Interventions, the Netherlands. DHvR has acted as a consultant for and received honoraria from Boehringer Ingelheim-Lilly Diabetes Alliance, Merck, Sanofi and AstraZeneca and has received research operating funds from Boehringer Ingelheim-Lilly Diabetes Alliance, AstraZeneca and Merck. All honoraria are paid to his employer. NMJH has received honoraria from Boehringer Ingelheim and Novo Nordisk. MRS is a scientific advisory board member of Clear.bio, the Netherlands. None of these interests are relevant to this article. The other authors declare that there are no relationships or activities that might bias, or be perceived to bias, their work.
