# Supplementary material for: Higher fibre and lower carbohydrate intake are associated with favourable CGM metrics in a cross-sectional cohort of 470 individuals with type 1 diabetes
Source: Diabetologia. 2024 Jul 5;67(10):2199–209. doi: 10.1007/s00125-024-06213-5 (PMC11446970; doi:10.1007/s00125-024-06213-5)
Supplement: Supplementary file 1 — Supplementary file1 (PDF 870 KB) [file 125_2024_6213_MOESM1_ESM.pdf]

ESM Table 1. Food intake in the total group (*n*=470) and stratified by TIR ≥ 70% or <70%. Data are presented as mean (SD).

	**Total (*n*=470)**	**TIR<70% (*n*=257)**	**TIR≥70% (*n*=213)**
**Daily**			
Energy, kJ	6878.50 (2001.10)	6751.94 (1957.40)	7031.21 (2046.78)
Carbohydrates, g	162.33 (62.58)	167.64 (57.49)	155.92 (67.80)
Fat, g	74.94 (30.83)	70.70 (27.88)	80.06 (33.39)
Protein, g	69.92 (23.81)	67.35 (21.50)	73.03 (26.05)
Fibre, g	20.24 (9.03)	18.73 (8.27)	22.07 (9.58)
Sugar, g	62.71 (31.65)	62.94 (30.71)	62.44 (32.82)
**Breakfast**			
Energy, kJ	1241.54 (674.21)	1232.93 (645.47)	1251.28 (706.97)
Carbohydrates, g	29.74 (18.23)	31.20 (17.64)	28.12 (18.80)
Fat, g	13.35 (11.51)	12.45 (10.69)	14.36 (12.32)
Protein, g	12.78 (7.80)	12.77 (7.85)	12.79 (7.76)
Fibre, g	4.28 (3.28)	4.04 (2.80)	4.56 (3.74)
Sugar, g	13.90 (11.72)	14.72 (11.74)	12.97 (11.67)
**Lunch**			
Energy, kJ	1748.30 (748.59)	1729.58 (803.46)	1769.52 (682.63)
Carbohydrates, g	39.30 (21.90)	41.53 (24.00)	36.80 (19.03)
Fat, g	19.35 (11.75)	17.83 (10.26)	21.05 (13.05)
Protein, g	19.09 (9.30)	18.28 (9.43)	19.99 (9.09)
Fibre, g	5.48 (3.08)	5.02 (2.87)	5.98 (3.24)
Sugar, g	11.00 (9.94)	11.42 (11.02)	10.53 (8.59)
**Dinner**			
Energy, kJ	2578.09 (1016.28)	2574.72 (1042.48)	2581.91 (988.51)
Carbohydrates, g	54.23 (27.32)	56.79 (26.52)	51.35 (27.99)
Fat, g	27.67 (14.43)	26.66 (14.00)	28.80 (14.85)
Protein, g	29.83 (14.00)	28.86 (13.92)	30.91 (14.06)
Fibre, g	7.71 (4.31)	7.17 (4.44)	8.32 (4.09)
Sugar, g	13.71 (9.20)	13.39 (9.46)	14.07 (8.91)
**Snacks**			
Energy, kJ	1641.75 (1151.61)	1606.94 (1190.71)	1681.19 (1107.50)
Carbohydrates, g	43.31 (36.26)	42.52 (32.26)	44.19 (40.34)
Fat, g	17.21 (14.57)	16.12 (14.13)	18.43 (14.99)
Protein, g	9.76 (9.45)	8.82 (8.76)	10.81 (10.08)
Fibre, g	3.88 (4.10)	3.27 (3.15)	4.56 (4.87)
Sugar, g	25.26 (21.21)	24.48 (20.45)	26.14 (22.06)

ESM Table 2. Spearman correlations between energy intake, macronutrients and CGM metrics.

	TIR	TBR	TAR	GCV
**ENERGY, KJ**	0.10*	-0.06	-0.08	-0.06
**CARBOHYDRATES**	-0.15*	0.01	0.16*	0.18*
**PROTEIN**	0.17*	-0.06	-0.16*	-0.14*
**FIBRE**	0.23*	-0.04	-0.22*	-0.16*
**FAT**	0.18*	-0.08	-0.17*	-0.16*
**SUGAR**	-0.04	0.01	0.05	0.12*

An asterisk represents a p-value <0.05.

ESM Table 3. Associations of energy intake and macronutrients with time in range.

TIR ≥70%				
	Model	OR	Lower CI	Upper CI
**Energy intake**	Crude	1.15	0.96	1.38
	1	1.16	0.94	1.42
**Carbohydrates**	Crude	0.82	0.67	0.99
	1	0.83	0.66	1.04
	2	0.63	0.48	0.82
	3	0.67	0.51	0.87
**Protein**	Crude	1.30	1.06	1.59
	1	1.29	1.03	1.63
	2	1.01	0.74	1.38
	3	1.03	0.75	1.42
**Fat**	Crude	1.41	1.15	1.75
	1	1.39	1.10	1.77
	2	1.21	0.88	1.68
	3	1.10	0.79	1.52
**Fibre**	Crude	1.52	1.24	1.89
	1	1.48	1.18	1.88
	2	1.69	1.26	2.29
	3	1.64	1.22	2.24
**Sugar**	Crude	0.98	0.81	1.19
	1	1.00	0.81	1.23
	2	0.85	0.67	1.07
	3	0.87	0.69	1.10

Odds ratios (95%CI) for TIR ≥70% are depicted per SD intake of each macronutrient. One SD represents 63 g carbohydrates, 31 g fat, 9 g fibre, 32 g sugar, or 2001kJ, respectively. Model 1[a]: Adjusted for age, sex, diabetes duration, socioeconomic status, BMI, pump use, exercise, alcohol intake. Model 2: model 1 + other macronutrients listed. Model 3: model 2 + daily insulin use.

Sugar and carbohydrates are not added in the same model, see method section ‘Data collection’. [a]

For energy intake, daily insulin use is added to model 1.

ESM Table 4. Associations of energy intake and macronutrients with time below range.

TBR (<4%)				
	Model	OR	Lower CI	Upper CI
**Energy intake**	Crude	1.16	0.95	1.42
	1	1.24	0.99	1.55
**Carbohydrates**	Crude	1.09	0.89	1.35
	1	1.25	0.99	1.60
	2	1.32	1.01	1.74
	3	1.34	1.02	1.78
**Protein**	Crude	1.11	0.9	1.39
	1	1.12	0.88	1.43
	2	1.02	0.73	1.43
	3	1.01	0.73	1.42
**Fat**	Crude	1.18	0.94	1.49
	1	1.17	0.92	1.51
	2	1.20	0.85	1.70
	3	1.21	0.85	1.73
**Fibre**	Crude	1.01	0.81	1.26
	1	1.02	0.80	1.30
	2	0.82	0.61	1.11
	3	0.80	0.60	1.09
**Sugar**	Crude	1.11	0.9	1.38
	1	1.19	0.96	1.50
	2	1.20	0.95	1.54
	3	1.21	0.95	1.56

Odds ratios (95%CI) for TBR <4% are depicted per SD intake of each macronutrient. One SD represents 63 g carbohydrates, 31 g fat, 9 g fibre, 32 g sugar, or 2001 kJ, respectively. Model 1[a]: Adjusted for age, sex, diabetes duration, socioeconomic status, BMI, pump use, exercise, alcohol intake. Model 2: model 1 + other macronutrients listed. Model 3: model 2 + daily insulin use.

Sugar and carbohydrates are not added in the same model, see method section ‘Data collection’. [a]

For energy intake, daily insulin use is added to model 1.

ESM Table 5. Associations of energy intake and macronutrients with time above range.

TAR (<25%)				
	Model	OR	Lower CI	Upper CI
**Energy intake**	Crude	1.22	1.02	1.47
	1	1.21	0.99	1.50
**Carbohydrates**	Crude	0.83	0.68	1.01
	1	0.83	0.66	1.03
	2	0.61	0.47	0.80
	3	0.65	0.49	0.85
**Protein**	Crude	1.37	1.12	1.68
	1	1.35	1.08	1.70
	2	1.00	0.73	1.37
	3	1.02	0.74	1.40
**Fat**	Crude	1.53	1.24	1.91
	1	1.51	1.20	1.92
	2	1.33	0.96	1.85
	3	1.29	0.92	1.81
**Fibre**	Crude	1.57	1.27	1.95
	1	1.52	1.21	1.92
	2	1.70	1.27	2.3
	3	1.65	1.23	2.24
**Sugar**	Crude	0.96	0.79	1.16
	1	0.97	0.79	1.19
	2	0.80	0.63	1.01
	3	0.82	0.65	1.04

Odds ratios (95%CI) for TAR <25% are depicted per SD intake of each macronutrient. One SD represents 63 g carbohydrates, 31 g fat, 9 g fibre, 32 g sugar, or 2001 kJ, respectively. Model 1[a]: Adjusted for age, sex, diabetes duration, socioeconomic status, BMI, pump use, exercise, alcohol intake. Model 2: model 1 + other macronutrients listed. Model 3: model 2 + daily insulin use.

Sugar and carbohydrates are not added in the same model, see method section ‘Data collection’. [a]

For energy intake, daily insulin use is added to model 1.

ESM Table 6. Associations of energy intake and macronutrients with glucose coefficient of variance.

Glucose coefficient of variance (<36%)				
	Model	OR	Lower CI	Upper CI
**Energy intake**	Crude	1.08	0.89	1.31
	1	1.11	0.89	1.38
**Carbohydrates**	Crude	0.80	0.65	0.97
	1	0.85	0.67	1.07
	2	0.68	0.51	0.90
	3	0.69	0.51	0.90
**Protein**	Crude	1.16	0.94	1.44
	1	1.18	0.93	1.52
	2	0.89	0.63	1.26
	3	0.88	0.62	1.25
**Fat**	Crude	1.38	1.10	1.75
	1	1.35	1.05	1.75
	2	1.36	0.95	1.98
	3	1.36	0.94	2.00
**Fibre**	Crude	1.34	1.08	1.68
	1	1.32	1.03	1.71
	2	1.48	1.08	2.07
	3	1.47	1.07	2.06
**Sugar**	Crude	0.9	0.74	1.1
	1	0.96	0.77	1.19
	2	0.83	0.66	1.06
	3	0.84	0.65	1.06

Odds ratios (95%CI) for GCV <36% are depicted per SD intake of each macronutrient. One SD represents 63 g carbohydrates, 31 g fat, 9 g fibre, 32 g sugar, or 2001 kJ, respectively. Model 1 [a]: Adjusted for age, sex, diabetes duration, socioeconomic status, BMI, pump use, exercise, alcohol

intake. Model 2: model 1 + other macronutrients listed. Model 3: model 2 + daily insulin use. Sugar and carbohydrates are not added in the same model, see method section ‘Data collection’. [a] For energy intake, daily insulin use is added to model 1.

ESM Table 7. Missing values per variable (total cohort, *n*=470).

Variable	Missing values (*n*)	Missing values (*%*)
Female	0	0
Age	0	0
Weight	0	0
BMI	0	0
Diabetes duration	0	0
Socieconomic status	16	3.4
Insuline	2	0.4
Alcohol use	0	0
Smoking	0	0
Medication other than insulin	0	0
Antiypertensive drugs	2	0.4
Anticoagulants	1	0.2
Thyroid medication	1	0.2
Other drugs	1	0.2
Sensor	18	3.8
Pump type	0	0
LDL	1	0.2
eGFR	2	0.4
HbA1c	2	0.4
TIR	0	0
TBR	0	0
TAR	0	0
GCV	36	7.7
Energy intake, kJ	0	0
Carbohydrate intake	0	0
Fat intake	0	0
Protein intake	0	0
Fibre intake	0	0
Sugar intake	3	0.6
